# Nanopore Sequencing for De Novo Bacterial Genome Assembly and Search for Single-Nucleotide Polymorphism

**DOI:** 10.3390/ijms23158569

**Published:** 2022-08-02

**Authors:** Maria G. Khrenova, Tatiana V. Panova, Vladimir A. Rodin, Maxim A. Kryakvin, Dmitrii A. Lukyanov, Ilya A. Osterman, Maria I. Zvereva

**Affiliations:** 1 Department of Chemistry, Lomonosov Moscow State University, 119991 Moscow, Russia; tvk@genebee.msu.ru (T.V.P.); rodinva@my.msu.ru (V.A.R.); dmitrii.lukianov@skoltech.ru (D.A.L.); i.osterman@skoltech.ru (I.A.O.); zvereva@genebee.msu.ru (M.I.Z.); 2 Bach Institute of Biochemistry, Federal Research Centre “Fundamentals of Biotechnology” of the Russian Academy of Sciences, 119071 Moscow, Russia; 3 School of Bioengineering and Bioinformatics, Lomonosov Moscow State University, 119991 Moscow, Russia; maxim.kryakvin@gmail.com; 4 Skolkovo Institute of Science and Technology, Center of Life Sciences, 121205 Moscow, Russia

**Keywords:** ONT sequencing, antibiotic resistance, *tolC* gene, SNV, deletion

## Abstract

Nanopore sequencing (ONT) is a new and rapidly developing method for determining nucleotide sequences in DNA and RNA. It serves the ability to obtain long reads of thousands of nucleotides without assembly and amplification during sequencing compared to next-generation sequencing. Nanopore sequencing can help for determination of genetic changes leading to antibiotics resistance. This study presents the application of ONT technology in the assembly of an *E. coli* genome characterized by a deletion of the *tolC* gene and known single-nucleotide variations leading to antibiotic resistance, in the absence of a reference genome. We performed benchmark studies to determine minimum coverage depth to obtain a complete genome, depending on the quality of the ONT data. A comparison of existing programs was carried out. It was shown that the Flye program demonstrates plausible assembly results relative to others (Shasta, Canu, and Necat). The required coverage depth for successful assembly strongly depends on the size of reads. When using high-quality samples with an average read length of 8 Kbp or more, the coverage depth of 30× is sufficient to assemble the complete genome de novo and reliably determine single-nucleotide variations in it. For samples with shorter reads with mean lengths of 2 Kbp, a higher coverage depth of 50× is required. Avoiding of mechanical mixing is obligatory for samples preparation. Nanopore sequencing can be used alone to determine antibiotics-resistant genetic features of bacterial strains.

## 1. Introduction

Genome assembly is one of the most important tasks in modern genomics. The most common pipeline involves variants of genome assembly by alignment of reads on the reference genome. However, in some cases a relevant reference genome is not known. In this case, a de novo genome assembly is required, involving the construction of a complete nucleotide sequence without a reference [1]. This task is extremely difficult if using next-generation sequencing (NGS), because the typical lengths of reads are hundreds of nucleotides long [2]. The development of nanopore sequencing (Oxford nanopore technology, ONT) [3] enables the possibility to obtain reads of several thousand base pairs and more, which facilitates genome assembly without a reference template [4]. However, the limitation of this methodology is the relatively high error range, which in some cases can typically reach up to 10%, produced in nucleotide resolution. In order to counter this setback, a hybrid genome assembly that combines NGS and nanopore sequencing data can be utilized [5].

The number of applications of ONT sequencing is rapidly growing [6,7,8,9,10]. One of the applications of biomedical chemistry is the study of various bacterial strains [11], including those exhibiting resistance to antibiotics [12], which occurs due to the evolution of point mutations of nucleotides or global rearrangements of the genome [13,14]. Our aim was to evaluate the applicability of ONT technology (without combination with NGS data) for de novo assembly and detecting genetic changes leading to antibiotic resistance, including global genomic rearrangements and point mutations.

In this paper, we present a benchmark study of the de novo genome assembly based only on the ONT data and investigate the applicability of available bioinformatics software tools. The quality control of de novo assembled genomes is performed by verification of the known deletion of a gene, as well as the search for individual substitutions of nucleotides in genes found in independent experiments. The DNA of a well-characterized bacterial strain JW5503 of *E. coli* [15] which exhibits a deleted *tolC* gene [16] obtained in five independent isolations, were used as samples for the study. The literature presents heterogeneous data on the required coverage depth when using ONT technology; therefore, we conduct a detailed comparison of the quality of samples, as well as the required coverage depth for the complete genome assembly and determination of the mechanism of antibiotic resistance.

## 2. Results and Discussion

### 2.1. Characterization of Samples

We started with a detailed analysis of the DNA samples considered for the study, as it is required for the subsequent benchmark study. Our main interest centered on the reliable determination of the SNVs as well as de novo genome assembly for analysis of the bacterial isolates that can be resistant to a range of antibiotics or produce new metabolites. Analysis of bacterial genomes reveal genomic changes resulting in antibiotic resistance mechanisms [17,18]. Determination of these mechanisms is one of the most important steps in particular, by antibiotic-producing strains or strains with the high susceptibility to antimicrobial activity exerted by novel drugs. Selection of resistant clones followed by genome sequencing can help to identify genes that are involved in interaction with an antibiotic. Mutations in genes involved in pivotal cellular processes—i.e., replication, transcription, translation or the formation of the cell wall—are typical targets affected by antibiotics, and may even indicate the target molecule. Several other attributes that render cells resistant to antibiotics, such as the efflux system, can expand our knowledge gap on how cells thrive under stress from small molecules. The main features of genomic changes are SNVs and rearrangements of the genome that alter the functioning of genes. We selected two unique changes: point substitutions and genomic rearrangement, characterized by an alternative experiment, to evaluate the efficiency of genome assembly based on nanopore sequencing data.

The analysis of five samples of genomic DNA (T1–T5) isolated from laboratory bacterial strains with known initial genome, possessing antibiotic resistance and characterized by a deletion of the *tolC* gene [16] was carried out. The main objectives of the work were to determine the necessary characteristics of the isolated DNA sample for the de novo assembly of the complete genome, the choice of the optimal bioinformatic tool, as well as the required coverage depth to determine point substitutions (Table 1) and genome rearrangements leading to antibiotic resistance.

**Figure 1:**
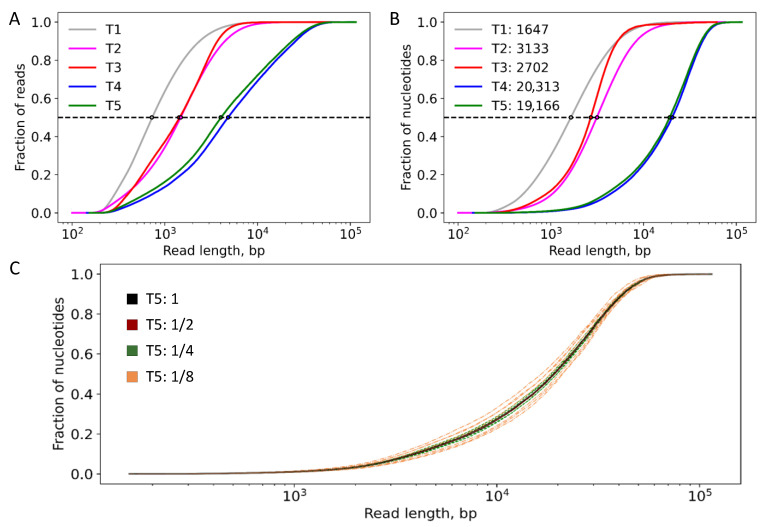
Accumulation curves demonstrating read length distributions for considered samples. (**A**,**B**) Entire samples T1–T5. Each point on the curve corresponds to the fraction of all (**A**) reads or (**B**) nucleotides, with the read length equal to or smaller than the read length at this point. In the legend of panel (**B**), the values after colons are the lengths of reads at which the curves reach the fraction value of 0.5. (**C**) The same graph as (**B**) obtained for the T5 set and its subsets, values after colons are the fractions of reads from the entire set of T5 sample in the corresponding curves.

The quality of the initial samples varies greatly, as can be seen from the main characteristics presented in Table 1. All available genomic DNA was used in the analysis and the input quality control of the nucleic acids for impurities was performed (optical density ratio A260/A280/A230). Only genomic DNA of sample T1 was additionally treated with MagAttract to get correct optical density ratio. Reagents for extraction are comparable for all samples. The analysis of length distribution of DNA fragments is shown in Figure 1. The most fragmented is the T1 sample. It is characterized by an average read length of slightly more than 1 Kbp (base pairs). Samples T2 and T3 are characterized by average read lengths that are approximately twice as large. T4 and T5 samples differ significantly with the average read length exceeding 8 Kbp. Pronounced differences in distributions of reads lengths in samples T1–T5 are explained by the following. T1 is maximally fragmented due to the additional cleaning step. The mechanical mixing (vortexing) in the process of genomic DNA isolation was utilized only during the preparation of T1–T3 samples. Evaluation of the mechanical mixing (vortexing) effect on DNA length described in Appendix A. In addition, the minimum possible mechanical action during the quality assessment protocol, gel electrophoresis, and DNA preparation to sequencing was applied for T4–T5 samples. The distribution of fragment lengths before nanopore sequencing can be estimated based on the analysis of gel electrophoresis data at the stage of assessing the quality of genomic DNA samples (Table 1). Taken together, the best result for sequencing can be achieved by minimizing vortexing of samples and mechanical shearing impact at all steps (not only during the isolation of genomic DNA, but also during sample preparation for sequencing) in order to maintain the integrity of the genome, which yields better resolution upon sequencing.

For all samples except T3, the coverage depth is significant and exceeds 100×. Also, the T1 sample is characterized by relatively small read lengths. Therefore, to perform benchmark studies, we chose T2, T4, and T5 samples and created subsets that conserve the main feature of the entire set—that is the read length distributions are the same for the entire set and its subsets (as shown as an example for sample T5 on Figure 1C). The parameter of interest, which we vary, is the coverage depth. The subsets include half, fourth, and eighth parts of the numbers of reads of the entire sets.

### 2.2. Genomic Rearrangements

**Figure 2:**
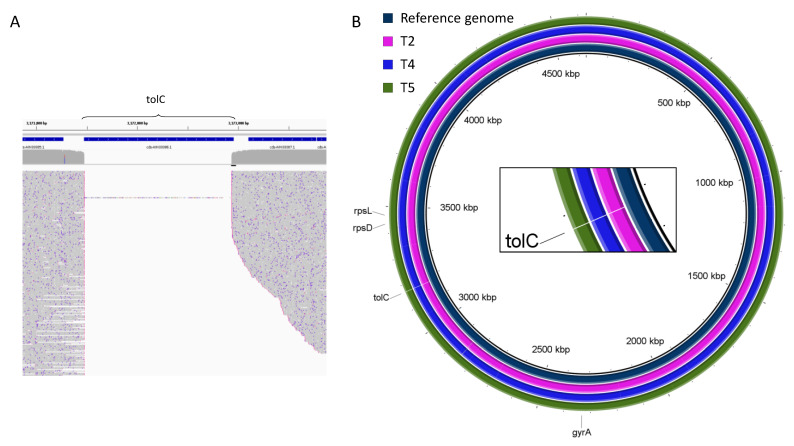
Deletion of the *tolC* gene. Alignment of (**A**) ONT reads to the reference genome of the *E. coli* (**B**) de novo assembled genomes on the reference genome of the *E. coli*. All genes that are considered in this study are marked.

The simplest way to find genomic rearrangement (gene deletions or insertions) is to align reads to the reference sequence. We utilized Samtools [19,20] and Minimap2 [21,22] programs for all considered samples and aligned ONT reads on a reference genome of *E. coli* BW25113 (GenBank: CP009273.1). We found the deletion of the *tolC* gene for all samples (see Figure 2A for example of T2 sample).

Several programs were used to assemble genomes de novo: Shasta [23], Canu [24], Necat [25] and Flye [26,27]. The quality control of genome assembly was carried out qualitatively according to the ability to organize fragments into a single ring fragment containing a deletion in the *tolC* gene region with the genome size same as the reference genome. Previous studies have shown that the de novo genome collection is performed more efficiently using the Flye program [26,27]. Furthermore, error correction was carried out by using the Medaka program [28]. According to the results of our simulations, this program turned out to be the only one that succeeded with the genome assembly for the T2 sample. It constructed a circular genome of 4.6 Mb. The same was observed for the T4 and T5 samples. The rest of the programs assembled sets of contigs, but not a single genome for T2 that was a complicated sample for the de novo genome assembly due to the relatively short read lengths, despite high coverage depth. The T3 sample genome assembly with Flye resulted in the construction of a set of 102 fragments with the size not exceeding 300 Kb and with the total length of all contigs being approximately 4.6 Mb—that is the size of the *E. coli* genome. Thus, the mean 25× coverage in T3 was not enough for the genome assembly.

In order to quantify the required coverage depth accurately, we performed an additional benchmark analysis. To do this, we obtained subsets of reads from T2, T4, and T5 samples, as described above. For example, the initial coverage depth for sample T2 was 417×. This made it possible to assemble a complete genome with a size of 4.6 Mb. Evaluation of the assembled genome was performed by the generation of a ring genome relative to the known reference genome (Figure 2B). When studying datasets of one-half of the original ones, the coverage depth was about 200×. This also enabled the assembly of complete genomes of 4.6 Mb. Further reduction of the data set to an average coverage of about 100x also made it possible to construct complete genomes. The following two-fold reduction of the dataset to an average coverage of about 50× made it possible to compose eight test samples characterized by similar parameters of distributions over read lengths. In two cases, complete 4.6 Mb genomes were assembled. However, in six other cases, four separate fragments with a length of the largest contig being 3.8 Mb were assembled for each dataset. When the dataset was further divided in half, the de novo genome assembly resulted in the formation of a large number (4 or more) of individual fragments with a maximum length of about 1 Mb.

The T4 and T5 also have high coverage, and the read lengths are about four times larger than for the T2 sample. Thus, we performed the same data partitioning for these samples to study the influence of the read length on the required coverage depth for the de novo genome assembly. The average coverage depth for the T4 and T5 samples was 144x and 181x, respectively. We obtained subsets with the half, fourth, and eighth parts of the entire number of reads and assembled complete genomes up to the mean depth of 15x. 

The *tolC* gene was not detected in samples including T2–T5 and their subsets. The important conclusion is that the required coverage depth for the de novo genome assembly strongly depends on the sample quality, that is for the set of longer reads a smaller coverage is required. For samples with the mean read length of about 8 Kbp, the 30× is enough, whereas for the lower quality samples with the 2 Kbp mean length the coverage depth should be increased to more than 50× when utilizing Flye [26,27]. To compare, a similar de novo bacterial genome assembly was performed by using Canu [24] and samples with the 4.5–7 Kbp mean lengths of reads with the coverage depth of 80×–240× [11]. The circular genome assembly was only in one of twelve cases; in others the set of contigs were obtained.

### 2.3. Single Nucleotide Variations

Determination of the single-nucleotide variations (SNVs) requires a more strict test that can be utilized to determine the quality of samples and assembled genomes. As a reference, we used experimentally scored SNVs in T3–T5 samples. These mutations include: *gyrA* gene in T3; *rpsL* gene in T4; *rpsL*, and *rpsD* genes in T5, which were independently scored by Sanger sequencing. SNV determination from ONT data was performed in two different ways. First, we utilized Medaka program [28] and aligned subsets of reads to the sequence of the corresponding gene (Table 2). This allowed us to find SNV, the coverage depth at the corresponding coordinate and error of the SNV estimates. Single substitutions were detected for most of the collected genomes. For datasets with larger than 30× coverage the error of the estimates did not exceed 3%. Alternative benchmark experiment was to locate the same SNVs in de novo assembled genomes (last column in Table 2). With respect to de novo assembled genomes, we found that single nucleotide substitutions can be confidently determined with a coverage depth of more than 30×.

To conclude, the SNV can be confidently determined with the error less than 1% if the coverage depth at this coordinate is 30× or more. Lower coverage leads to the larger possible errors in the SNV determination or false negative or false positive SNVs.

## 3. Materials and Methods

### 3.1. Characterization of Strains

The kanamycin-resistant *E. coli* ΔtolC (*E. coli* JW5503) strain was obtained from the Keio collection [16]. The isogenic strain with a P90L substitution in S12 ribosomal protein was obtained as a result of streptomycin-resistant clone search by spontaneous mutagenesis; this strain is streptomycin-depended [29,30]. Then, a streptomycin-resistant revertant strain [31] with P90L substitution in the S12 ribosomal protein as in the initial strain and with the I199N substitution in S4 was selected from the streptomycin-dependent strain by spontaneous mutagenesis. Mutation in S12 protein was confirmed by Sanger sequencing of the *rpsL* gene (S12seqF ACGTGTTTACGAAGCAAAAGC, S12seqR AGTTTGACATTTAAGTTAAAACG). Mutation in S4 protein was confirmed by Sanger sequencing of the *rpsD* gene (S4seqF CAGATGCTGCCCGTGAAG, S4seqR CAGACGACCGATTGCACTG).

### 3.2. Resistant Clones Obtaining by Spontaneous Mutagenesis

The selection of resistant clones was carried out in the LB agar medium with the addition of the antibiotic under investigation in concentrations of 2×, 3×, and 5× of the minimal inhibitory concentration (MIC). The cell cultures were grown on LB-agar with appropriate antibiotics for 18–72 h at 37 °C until single colonies were observable. Subsequently, MICs for the antibiotic investigated and erythromycin was further determined. Clones that exhibited a higher MIC for the test antibiotic, with no apparent change exerted in the erythromycin control, were used for whole-genome sequencing.

### 3.3. Minimal Inhibition Concentration Measurement

MIC measurements were conducted in 96-well plates. Rows 1 to 11 were loaded with *E. coli* JW5503 cell suspension obtained by dilution of the overnight culture 200 times. Cells (200 µL) were added to the first row, and 100 µL were added to the subsequent rows. The twelfth row was loaded with LB broth without cells as a control.

A volume of 4 µL of test samples were further added to the wells in the first row, followed by a gently mix and a subsequent two-fold serial dilution of the mixture. Erythromycin (2 µL from a stock of 5 mg/mL) was used as a control of the experiment. Then the plates were incubated at 37 °C with aeration (200 rpm) overnight. The cell concentration was determined by the optical density value, A600, measured by using the plate reader Victor X5 2030 (Perkin Elmer). The lowest concentration at which the test substance fully inhibited bacterial growth was scored as the respective MIC.

### 3.4. Genomic DNA Extraction

Bacterial genomic DNA samples were extracted by using the GeneJET Genomic DNA Purification Kit (Thermo Scientific) with a vortex mixer (samples T1 and T2) and LumiPure genomic DNA from AnySample Kit (Lumiprobe) with gentle mixing (samples T3–T5). For gentle mixing, we just manually inverted tube with the sample.

### 3.5. DNA Quality Control after Purification

DNA quality was evaluated by electrophoresis on 1% agarose gel and additionally by the ratios of absorbance at 260 nm and 280 nm, and at 260 nm and 230 nm by using a Nanodrop spectrophotometer (Thermo Scientific). According to the manufacturer’s recommendation, the A260/A280 acceptable ratio is 1.8–2.0, and the A260/A230 acceptable ratio is 2.0–2.2 for a DNA sample of good quality for the nanopore analysis. If the samples did not meet the given ratios, additional purification with MagAttract HMW DNA Handbook (Qiagen) kit was performed. The DNA concentration was then further scored by using the Qubit 3.0 fluorometer (Invitrogen, Thermo Fisher Scientific). Sample preparation for fluorometry was performed by using the Qudye dsDNA HS Assay kit (Lumiprobe).

Fragment lengths detection was performed on 1% agarose gel with 70 µg/mL of ethidium bromide by using a ChemiDoc scanner (Bio-Rad). Data analysis with the determination of the average fragment length was performed by using ImageLab v6.1 2020 by Bio-Rad Laboratories software.

### 3.6. Nanopore Sequencing

Nanopore sequencing was performed by Oxford Nanopore sequencing technology for genomic DNA by ligation by using the SQK-LSK109 protocol (OxfordNanopore). In the first step, DNA repair and end preparation for the adapter ligation were performed. The DNA control sample (DCS, OxfordNanopore) was thawed at room temperature, centrifuged, mixed by pipetting, and placed on ice. The NEBNext FFPE Repair Mix (NEB) and NEBNext Ultra II End repair/dA-tailing Module (NEB) reagents were prepared in accordance with manufacturer`s instructions. DNA tested samples were diluted with nuclease-free water according to the protocol. A total of 200 fmoles of genomic DNA were used for the OxfordNanopore R9.4.1 flow cell.

The DNA sample volume was adjusted to 45 µL with nuclease-free water in 1.5 mL Eppendorf DNA LoBind tubes, mixed thoroughly by flicking the tube (to avoid DNA fragmentation), and spun down. In a 0.2 mL thin-walled PCR tube, 1 µL of control DNA, 47 µL of tested DNA sample, 3.5 µL of NEBNext FFPE DNA Repair Buffer, 2 µL of NEBNext FFPE DNA Repair Mix, 3.5 µL of Ultra II End-prep reaction buffer, and 3 µL of Ultra II End-prep enzyme mix were combined. After gentle mixing by flicking the tube, it was spun down and incubated for 5 min at 20 °C following with incubation for 5 min at 65 °C. Then, the sample purification by using AMPure XP beads (Beckman Coulter) was performed. AMPure XP beads were resuspended by vortexing, and the DNA sample was transferred into a 1.5 mL Eppendorf DNA LoBind tube. A total of 60 µL of resuspended beads were added, the sample was mixing by flicking the tube, and incubated on a Hula mixer for 5 min at room temperature. For the washing step, 70% ethanol solution was prepared by using nuclease-free water. The sample was spun down and placed on a magnet unit until a pellet was formed and with the eluate clear and colorless. Although the tube is on a magnet unit, the supernatant was pipetted off, and the pellet was washed twice with 200 µL of freshly prepared 70% ethanol. After a brief spin down, the sample was placed on a magnetic unit, residual ethanol was pipetted off, and the pellet was dried for about 30 sec. Then the pellet was resuspended in 61 µL of nuclease-free water for 2 min at room temperature, placed on a magnetic unit, and clear and colorless DNA sample was separated from the pellet. The DNA sample (1 µL) was quantified by using a Qubit 4 fluorometer. Sample preparation for fluorometry was performed by using Qudye dsDNA HS Assay kit (Lumiprobe).

After DNA repair and end preparation step, the adapter ligation step was performed. AMX adapter mix (OxfordNanopore) and Quick T4 Ligase (NEB) were spun down and placed on ice. The ligation buffer (LNB, OxfordNanopore), elution buffer (EB, OxfordNanopore), and long fragment buffer (LFB, OxfordNanopore) was thawed at room temperature, spun down, and mixed by pipetting. A volume of 60 µL of DNA sample, 25 µL of LNB, 10 µL of Quick T4 Ligase, and 5 µL of AMX adapter mix were combined, gently mixed by pipetting, spun down, and incubated for 10 min at room temperature. The mixture was purified by using AMPure XP beads; 40 µL of resuspended beads were added to the DNA sample, mixed by pipetting and incubated for 5 min on a Hula mixer at room temperature, then spun down and placed on a magnet unit. After pellet formation, the supernatant was pipetted off. The pellet was washed with 200 µL of LFB, then the beads were resuspended by pipetting and placed on a magnet for a pellet formation. A clear and colorless supernatant was removed and the washing procedure was repeated. After the second washing with LFB, the sample was dried for 30 s, resuspended in 15 µL of EB and incubated for 10 min at room temperature. Then the sample was placed on a magnetic unit to form a pellet until the supernatant is clear and colorless. A total of 15 µL of the sample were retained in a 1.5 mL DNA LoBind tube and 1 µL was quantified by using a Qubit 4 fluorometer. The prepared DNA library was then stored on ice until it was loaded into a cell.

For the flow cell loading procedure, the sequencing buffer (SQB), loading beads (LB), flush tether (FLT), and flush buffer (FB) all from OxfordNanopore, were thawed at room temperature and placed on ice. SQB, FB, and FLT were mixed by vortexing and spun down. The DNA library was then loading on a R9.4.1 MinION Mk flow cell (OxfordNanopore) in accordance with the manufacturer`s protocol. 

Three independent sequencing runs and data collection procedures were performed for the analysis of five samples: the first one for the T1 sample, the second one for the T2 sample, and the third one for the T4 and T5 samples. During the sample preparation for the third run, all DNA samples mixing was performed as gently as possible (by smooth turning). 

For the data collection for several strains in a single run, the additional barcoding step was performed before the adapter ligation procedure by using a Native Barcoding Expansion 1–12 (EXP-NBD104) kit (OxfordNanopore). The barcoding step was performed according to the manufacturer`s protocol, which is similar to the adapter ligation step and includes the barcode ligation and DNA purification steps. After barcoding ligation, equimolar mixture of DNA samples was used in the adapter ligation step.

### 3.7. Data Analysis

We utilized a Guppy basecaller 5.0.17 [32] to convert raw data in fast5 format to the basecalled data in fastq format. Debarcoding of samples was performed with the same software together with the basecalling procedure. All reads with the quality Q < 7.5 were excluded from the subsequent data analysis. Four samples (all except T3) provided the coverage depth larger than 100x and three of them (T2, T4–T5) have relatively long reads. To perform benchmark studies, we obtained subsets with the 1/2, 1/4 and 1/8 fractions of the total amount of reads from T2, T4 and T5 samples. Thus, we got 2 subsets with 1/2 of the total amount of reads, 4 subsets with 1/4 of the total amount of reads and 8 subsets with 1/8 of the total amount of reads for each sample. These subsets were utilized to determine the coverage depth that is required for the genome assembly and single nucleotide variant determination. For de novo genome assembly, we utilized the Shasta [23], Canu [24], Necat [25] and Flye [26,27] programs. Genome polishing was performed with the Medaka [28]. Alignment to the reference genome was performed with the Samtools [20] and Minimap2 [21,22]. Visualization was performed in the IGV [33] and BRIG [34] by using the reference genome *E. coli* BW25113 (GenBank: CP009273.1). For SNV determination, Medaka [28] and GSAlign [35] were utilized.

## 4. Conclusions

We present a systematic study of de novo genome assembly. We control the quality of assembled genomes as well as reads by the ability to reproduce SNVs and deletion of gene found in alternative experiments for the same samples. Reads lengths strongly depend on the methodology of genomic DNA isolation and sample preparation; mechanistic impact should be minimized during sample preparation. This crucially affects the quality of the de novo genome assembly. Samples with the mean read length less than 1.5 Kbp can be hardly utilized for de novo genome assembly even if the high coverage depth can be provided. Among bioinformatic tools for de novo genome assembly, we mostly recommend the Flye program [26,27]. When using high-quality samples with an average read length of 8 Kbp or more, an average coverage of 30x is sufficient to assemble a complete genome by using the Flye program and reliably determine SNVs and deletions. If the reference genome is known, the 30x depth is enough to reliably determine SNVs by alignment to the reverence sequence. A methodological protocol for sample preparation and data analysis is proposed. It allows one to study antibiotic resistance of strains and metabolites by producing strains without hybrid assembly with the NGS data [36,37,38] which considerably lowers the cost.

## Figures and Tables

**Table 1:** Main features of the considered samples. Deletions and SNVs are confirmed by alternative experimental methods.

Sample	T1	T2	T3	T4	T5
Deletion	*tolC*	*tolC*	*tolC*	*tolC*	*tolC*
Gene with SNV	*-*	*-*	*gyrA*	*rpsL*	*rpsL*, *rpsD*
Total bases, Mbp	4377.9	1952.1	114.8	663.5	831.7
Number of reads	3,888,954	935,363	62,748	70,397	97,636
Mean coverage	952×	424×	25×	144×	181×
Mean read length (ONT data), bp	1126	2087	1829	9425	8518
Mean read length (gel electrophoresis), bp	12,000	12,759	12,916	13,244	13,255

**Table 2:** SNVs in samples T3–T5. The probability of error is determined by the quality of the data in this position and the coverage depth during the analysis by the Medaka program [28]. The Fraction column shows the fraction of reads from the entire set utilized for analysis. The values of the coverage depth and error probability are separated by commas if several subsets of data were generated with a given fraction of the entire sample. “-” refers to samples in which no SNV was found. The last column presents data on SNVs detection in de novo assembled genomes.

Sample	Gene	SNV and Its Coordinate	Fraction	Coverage Depth (Error Probability)	SNV in De Novo Genomes, Yes/No
T3	*gyrA*	248: C → T	1	21× (0.01%)	1/0
T4	*rpsL*	272: C → T	1	150× (0.5%)	1/0
			1/2	92× (3%), 58× (1%)	2/0
			1/4	50× (0.2%), 42× (0.7%), 31× (1%), 27× (0.3%)	3/1
			1/8	27× (0.3%), 23× (1%), 22× (2%), 20× (3%), 16× (5%), 15× (0.6%), 9× (28%),-	5/3
T5	*rpsD*	599: T → A	1	165× (0.001%)	1/0
			1/2	91× (0.006%), 74× (0.002%)	2/0
			1/4	53× (0.01%), 45× (0.002%), 38× (0.01%), 29× (0.02%)	4/0
			1/8	26× (0.01%), 24× (0.01%), 17× (0.01%), 21× (0.004%), 15× (0.02%),-,-,-	8/0
T5	*rpsL*	272: C → T	1	171× (0.4%)	1/0
			1/2	96× (1%), 75× (0.3%)	2/0
			1/4	50× (0.6%), 46× (2%), 41× (3%), 33× (3%)	4/0
			1/8	27× (5%), 27× (1%), 25× (2%), 23x (2%), 20× (5%), 19× (45%), -, -	5/3

## Data Availability

Not applicable.

## Appendix A

DNA purification was held with LumiPure genomic DNA from AnySample Kit (Lumiprobe). Steps with mechanical mixings (vortexing) were carried out with different time intervals of 0 (sample was mixed gently without vortexing), 5, 10 and 30 s on FVL-2400N (Biosan). For gentle mixing we just manually inverted tube with sample. The overnight culture was grown in separate tubes, then it was mixed all together in one tube and after that, it was transferred to Eppendorf tubes for harvesting. With modifications in mixing, the protocol was following.

Harvest bacteria were grown in a liquid culture medium by centrifugation at 5000 *g* for 5 min. Remove the supernatant. Resuspend the cell pellet in 100 μL of PBS. Transfer the cell suspension to a clean 1.5 mL microcentrifuge tube.

1. Set a heating block to 55 °C, and place the vial with Elution Buffer in the heating block.
2. Add to the sample
  - 400 μL of Lysis Solution AS,
  - 5 μL of RNase A,
  - 10 μL of Proteinase K. Mix well.
3. Incubate the tubes at 55 °C for 10 min with vortexing 2 times:
  - 0 s (samples were mixed gently without vortexing),
  - 5 s,
  - 10 s,
  - 30 s.
4. Add 300 μL of Sorption Solution to the sample. Mix 2 times:
  - 0 s (samples were mixed gently without vortexing),
  - 5 s,
  - 10 s,
  - 30 s.

1. Place a spin column in a collection tube. Add the lysate to the column. Centrifuge the column for 45 s. Discard the collection tube and place the spin column into a clean collection tube.
2. Add 500 μL of Wash Solution A, centrifuge the column for 30 s. Discard the collection tube and place the spin column into a clean collection tube.
3. Add 500 μL of Wash Solution B, centrifuge the column for 3 min. Discard the collection tube.

After that DNA concentration was measured on a nanodope. DNA length was estimated on the gel in three repeats each containing 200ng (Table A1). According to the gathered data, we can conclude that DNA extraction without mechanical mixing (vortexing) leads to longer DNA fragments.

**Table A1:** Main features of the considered samples.

Sample Vortexing Time	0 s	5 s	10 s	30 s
Mean read length (gel electrophoresis), bp	13,101.7 ± 72.4	12,851.1 ± 167.5	13,021.5 ± 51.4	12,942.5 ± 121.1
